# *‘Huang Qi Elixir’* for proteinuria in patients with diabetic nephropathy: a study protocol for a randomized controlled pilot trial

**DOI:** 10.1186/1745-6215-14-223

**Published:** 2013-07-18

**Authors:** Xiang Tu, Fang Liu, James B Jordan, Xue Feng Ye, Ping Fu, Fei Wang, Sen Zhong

**Affiliations:** 1 National Traditional Chinese Medicine Clinical Research Base/Teaching Hospital of Chengdu University of Traditional Chinese Medicine (Traditional Chinese Medicine Hospital of Sichuan Province), Chengdu, Sichuan Province 610072, China; 2 Division of Nephrology, West China Hospital of Sichuan University, Chengdu, Sichuan Province 610041, China; 3 Private Practice, 453 Cerrillos Rd., Bldg. E, Santa Fe, NM 87501, USA; 4 Department of Nephrology, Teaching Hospital of Chengdu University of Traditional Chinese Medicine (Traditional Chinese Medicine Hospital of Sichuan Province), Chengdu, Sichuan Province 610072, China; 5 Department of Gerontology, Teaching Hospital of Chengdu University of Traditional Chinese Medicine (Traditional Chinese Medicine Hospital of Sichuan Province), Chengdu, Sichuan Province 610072, China

**Keywords:** Traditional Chinese Medicine, Huang Qi Elixir, Diabetic nephropathy, Proteinuria, Clinical trials, Pilot trial

## Abstract

**Background:**

Diabetic nephropathy (DN) is the major complication of diabetes; proteinuria is the hall mark of DN. Currently, the treatment for proteinuria is mainly limited to angiotensin converting enzyme (ACE) inhibitors or angiotensin II receptor blockers (ARBs). According to Traditional Chinese Medicine (TCM) theory, Chinese medicinals ‘securing essence and tonifying the kidney’ may be appropriate for proteinuria. The most promising Chinese medicinals and formulae are introduced in the present study to form a potent formula for DN proteinuria. To make oral administration convenient, the formula will be processed in the form of granules.

**Methods/design:**

A randomized, multi-center pilot trial will be conducted. Forty eight participants with DN will be randomly assigned to one of four treatment groups:

1. A granule group, at 10 grams, three times daily (G10 group, n = 12);

2. A granule group, at 20 grams, three times daily (G20 group, n = 12);

3. A decoction group (D group, n = 12); and

4. An irbesartan group (Aprovel group, n = 12).

The following outcome measures will be used: the percentage change of the albumin-to-creatinine ratio; and the changes in serum creatinine, glomerular filtration rate, fasting plasma glucose and hemoglobulin from baseline to the end of the trial.

**Discussion:**

It is notable that most published clinical trials which assessed the efficacy of TCM on DN were of poor methodology and, therefore, their results have been invalidated. It is necessary to carry out well-designed clinical trials to provide sound evidence. The present trial is a study with potentially great value, for it will provide the parameters for future randomized, placebo-controlled, clinical trials with large sample sizes.

**Trial registration:**

The trial is registered on the Chinese Clinical Trial Registry: ChiCTR-TRC-12002718 (http://www.chictr.org/cn/proj/show.aspx?proj=3820).

## Background

The prevalence of diabetic nephropathy (DN) in China is not very clear; however, in Beijing, the city which has the best medical service in China, the incidence of DN in patients with type 2 diabetes is 35.7% and that of microalbuminuria is 13.6% [1].

In Traditional Chinese Medicine (TCM) theory, the kidney is a paramount organ. It stores essence and the essence transforms *Qi* and produces blood. In addition, the kidney is the source of genuine *Yin* and genuine *Yang*. It is called ‘the prenatal base of life’ in TCM textbooks [2]. In the TCM system, there are many Chinese medicinals classified as tonifying and replenishing medicinals. Many of them have the effects of replenishing *Qi* of the kidney or tonifying kidney *Yang*.

We have known that DN is the major complication of diabetes and proteinuria is the hallmark of DN. DN is also defined by increased urinary albumin excretion (UAE) in the absence of other renal diseases, which is categorized into stages according to the level of UAE, that is, microalbuminuria (30 to 299 mg/24 hours) and macroalbuminuria (300 mg/24 hours or greater) [3]. Currently, the treatment for proteinuria is largely limited to angiotensin converting enzyme (ACE) inhibitors or angiotensin II receptor blockers (ARBs) [4]. Although the clinical beneficial effects of many ACE inhibitors or ARBs have been established [5], the efforts of looking for new drugs for DN that involve reducing proteinuria never stop. For example, many researchers investigated astragalus for the treatment of DN and the results seemed intriguing. Zhang et al. [6] systematically reviewed published animal experiments that had assessed the renal protective effects of astragalus in diabetic rat models. Their results showed significant beneficial effects of astragalus involving improved fasting blood glucose levels, glomerular filtration rate (GFR), urinary albumin excretion rate, and thickness of the glomerular basement membrane. Hong and co-workers [7] reported that astragalus might ‘play protective roles in diabetic nephropathy through multiple pathways at the gene level’.

Is TCM really able to lower proteinuria in patients with DN? Current evidence has tried to address this issue, but the picture remains unclear [8]. The present research looks at the DN-lowering proteinuria effect of TCM to answer this question. To evaluate the effects of TCM accurately, the most promising Chinese medicinals and formulae are used in the present study to form a potent formula for the treatment of proteinuria of DN.

Because proteinuria, in TCM theory, results from the leakage of the essence of the human body due to an insufficiency of the kidney, it is promising to look for Chinese medicinals that secure the essence and tonify the kidney for the treatment of proteinuria. A typical herb that secures essence and tonifies the kidney is used in the present study, Flastem Milkvetch Seed. A famous classical formula securing the essence and tonifying the kidney is also introduced in the present study for the treatment of proteinuria (Water-land Two Elixirs). It consists of two Chinese medicinals, Gordon Euryale Seed and Cherokee Rose Fruit. The first ingredient is grown in water and the second one is grown on land, which is the reason why it is named ‘Water-land.’ This formula is very effective for treating conditions such as spermatorrhea and enuresis [9]. However, only securing essence is not enough; because *Yin/Yang* has been damaged, it is essential to repair the balance between them.

There is a therapeutic strategy in TCM theory, namely, using ‘cool’ and ‘warm’ herbs together to balance *Yin/Yang*; this strategy is introduced in the present study. As the most conspicuous topic in the area of TCM treatment for DN, astragalus may be a very potent Chinese medicinal for proteinuria; therefore, it is also used in the present study. Both basic and clinical studies have demonstrated that astragalus could exert beneficial effects on DN [10-12]. An *in vitro* study investigated the effect of calycosin and calycosin-7-O-β-D-glucoside. These two major isoflavonoids in astragalus were tested with high glucose-induced rat mesangial cell proliferation and advanced glycation end product (AGE)-induced human glomerular endothelial cell apoptosis. The results suggested that both isoflavonoids had a significant therapeutic potential to modulate the development and/or progression of DN [10]. Zhang and his co-workers studied the effects of astragalus polysaccharide (APS), an aqueous extract from the astragalus membranaceus roots, on gene expressions of nuclear factor-kappaB (NF-kappaB) and an inhibitory protein of NF-kappaB (IkappaB) in experimental DN rats induced by streptozotocin. The results showed that APS improved proteinuria and renal function and that the mRNA level of NF-kappaB in renal cortex was decreased and IkappaB mRNA expression was raised by APS [11]. A randomized controlled trial showed that astragalus, in combination with captopril, could significantly reduce UAE [12]. However, astragalus is an herb of warm properties and has the capacity to upset the balance of *Yin/Yang*. Coptidis, is an herb of cool properties and is used to counteract the potential harm caused by astragalus. Coptidis is used for proteinuria because not only does it have centuries of practice-based recognition as a reliable TCM remedy for diabetes mellitus but its potential as a treatment for DN has been established in recent evidence-based supportive research. Liu et al. [13] evaluated the effects of compound Rhizoma Coptidis capsule (CRCC) on early DN in rats induced by streptozotocin. Their results showed that CRCC could reduce the levels of fasting plasma glucose (FPG), blood urea nitrogen (BUN), serum creatinine (Scr), proteinuria, the expression of transforming growth factor-beta1 and IV-C proteins, and alleviate pathological lesions in renal tissue of diabetic rats with nephropathy.

As a result, five Chinese medicinals are used in the present study for the treatment of proteinuria: astragalus, coptidis, Flastem Milkvetch Seed, Gordon Euryale Seed and Cherokee Rose Fruit (the Latin name and English name of the Chinese herbs mentioned in the article are shown in Table 1).

**Table 1:** **Traditional Chinese Medicinal Herbs used in *Huang Qi Elixir *formula/granule**

**PinYin name**	**Latin name**	**English name**	**Daily decoction dose**	**Dose per 1,000 g granule**
Huangqi	*Radix Astragali*	Astragalus/Astragalus membranaceus	30 g	500g
Shayuanzi	*Semen Astragali Complanati*	Flastem Milkvetch Seed	30 g	500g
Huanglian	*Rhizoma Coptidis*	Golden Thread	10 g	167g
Jinyingzi	*Fructus Rosae Laevigatae*	Cherokee Rose Fruit	15 g	250g
Qianshi	*Semen Euryales*	Gordon Euryale Seed	15 g	250g

One of the most obvious inconveniences of TCM is the preparation. It is very difficult to ask patients to decoct TCM several times daily for a long time and, therefore, this research group decided to make the formula in the form of granules (the ingredients are shown in Table 1). The granules are processed using the following methods: first, *coptidis* is ethanol extracted; second, the other five herbs are decocted together and then aqueous extracted; third, the concentrate from the previous two steps is mixed and then spray-dried. The granule is a 10 g/package.

### Objective

The aim of this clinical study is: (1) to evaluate if the efficacy of *Huang Qi* Elixir for proteinuria in patients with microalbuminuria is equivalent to that of the decoction; (2) to establish the working dose of the TCM granule; and (3) to evaluate the difference in efficacy between the TCM granule and the ARB (that is, irbesartan).

## Methods/design

### Design

**Figure 1:**
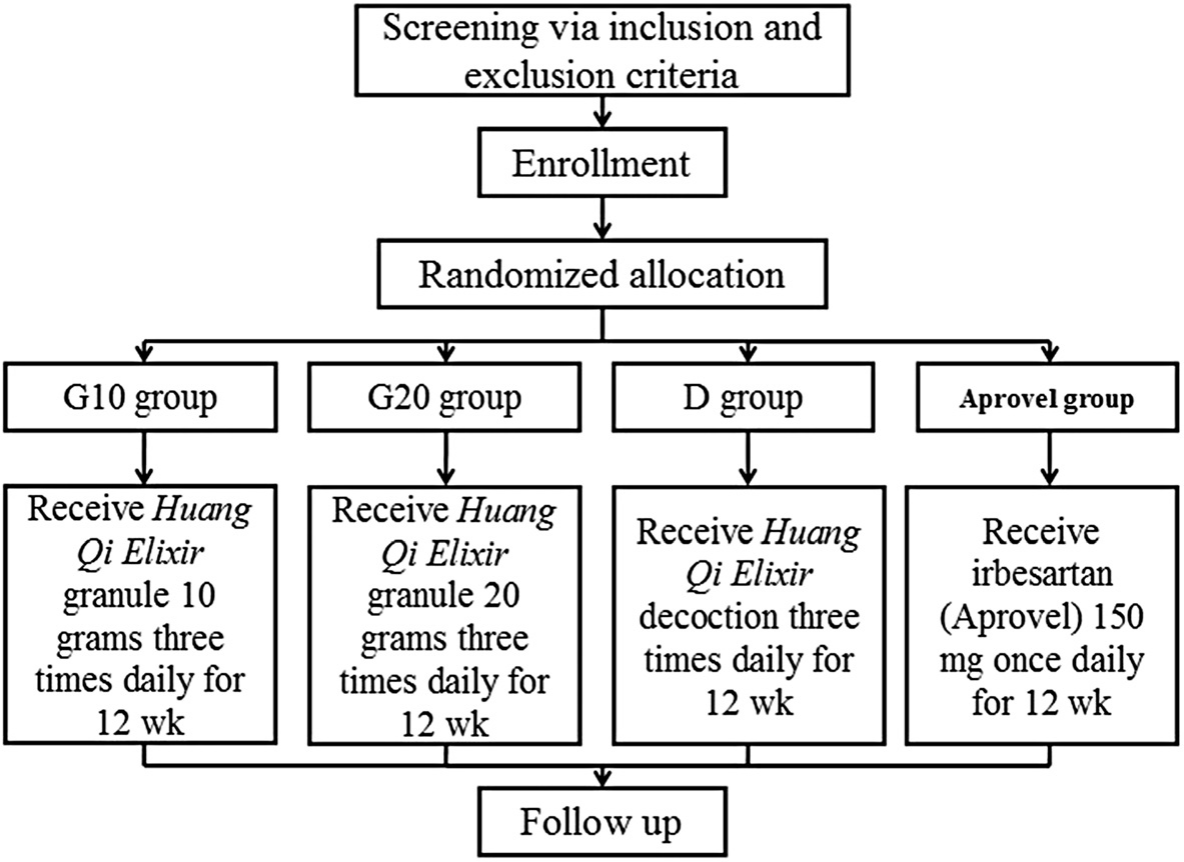
Flow chart of the clinical study.

This pilot study is a randomized, controlled, multi-center, clinical trial. The study will be sequentially conducted as follows: enrollment after screening via inclusion and exclusion criteria; randomization; a treatment period of 12 weeks; and assessment. The flow chart of the study is shown in Figure 1.

### Participants and eligibility

### Inclusion criteria

To be included in the study, patients must have:

1. symptoms of diabetes and a casual plasma glucose ≥11.1 mmol/L or fasting plasma glucose ≥7.0 mmol/L or in a oral glucose tolerance test, two-hour plasma glucose ≥11.1 mmol/L;

2. 30 mg/g < albumin-to-creatinine ratio (ACR) <300 mg/g or 30 mg/24hour <24 hour UAE <300 mg/24 hour, and GFR ≥40ml/min;

3. an age of 18 to 75 years;

4. acceptable glycemic control (FPG ≤10.0 mmol/L or postprandial plasma glucose ≤12 mmol/L and HbA1c ≤8.5%);

5. tolerablity to ARBs and blood pressure (BP) <150/90 mm Hg;

6. plasma albumin ≥30g/L

7. provided written informed consent.

The first two criteria must be met simultaneously.

### Exclusion criteria

Patients will be excluded if they have any of the following:

1. diabetes mellitus concurrent with non-diabetic kidney disease or are kidney transplant recipients;

2. concurrent severe disorders of heart, brain, liver or the hematopoietic system;

3. newly diagnosed or relapsed tumors;

4. mental disorders;

5. intolerance to oral medications or severe malabsorption;

6. hepatic dysfunction or a value of transaminase higher than one and a half times the upper limit;

7. allergy to TCM/ARBs or other allergies;

8. uncontrollable urinary infection or infections which influence the measurement of urinary protein;

9. pregnancy or lactation;

10. participation in other clinical trials within the past thirty days; and

11. anticipated survival <1 year.

### Recruitment

There are six centers involved in this clinical trial and participants will be recruited via posters at each participating center.

### Randomization and intervention

The study has four arms. After enrollment, 48 participants who meet the inclusion criteria will be randomly assigned to either: 1) granule group, 10 grams three times daily (G10 group, n = 12); 2) granule group, 20 grams three times daily (G20 group, n = 12); 3) the decoction group (D group, n = 12); or 4) the irbesartan group (Aprovel group, n = 12). The treatment course is 12 weeks. The randomization sequence will be generated with an SAS software package by the Good Clinical Practice (GCP) Center of the Teaching Hospital of Chengdu University of TCM and will be concealed and disseminated using opaque envelopes.

### Outcome measures

#### Primary outcome

The primary outcome, percentage change in ACR from baseline, will be measured at week 12. ACR will be measured with a spot urine test, every four weeks; 15 to 20 ml spot urine samples will be collected at each visit to assess albuminuria.

#### Secondary outcomes

The secondary outcomes include changes in GFR, Scr, HbA1c, urinalysis and FPG. All of these will be measured every four weeks except for HbA1c, which will be measured at week 12 only.

### Statistical methods

Statistical analyses will be conducted based on an intention-to-treat population. Participants who take at least one dose of drug and have one value on treatment comprise the full-analysis set (FAS) and those who complete a 12-week treatment comprise the per-protocol set (PPS). Missing data will be imputed with the use of the last-observation-carried-forward method, whereby missing values will be replaced by the last non-missing value. Repeated measures and multivariate analysis of variance of the general linear model will be applied to determine the changes in GFR, Scr and FPG at each visit. Percentage reduction from baseline in ACR and changes from baseline in HbA1c will be summarized and comparisons will be made by using a one-way analysis of variance.

### Compliance

Establishment of TCM and irbesartan after each visit will be quantified in order to enhance medication compliance. Participants whose compliance with TCM or irbesartan is < 80% of the total dose will be considered to have dropped out.

### Adverse events

All adverse events related to TCM and irbesartan will be reported to the ethics committee of the Teaching Hospital of Chengdu University of Traditional Chinese Medicine and also to each participating center in written case report form. Safety will be monitored using routine blood examination, liver and renal function, blood electrolytes and electrocardiogram (ECG). Other potential adverse events will be clearly documented and followed up until they are resolved or alleviated.

### Sample size

Each arm will only include 12 subjects, for this is just a pilot trial aimed at exploring some parameters for a future clinical trial with a large sample size.

### Ethics

The study protocol and the written informed consent were approved by the Sichuan Regional Ethics Review Committee on Traditional Chinese Medicine (2012KL-013). Written informed consent will be obtained from each patient.

## Discussion

Many clinical trials have been carried out to evaluate the efficacy of TCM for DN [14,15]. Liu et al. [16] systematically reviewed randomized controlled trials evaluating the efficacy and safety of astragalus in the treatment of DN. Their results included 33 RCTs and one quasi-RCT involving 2,356 patients and showed that it ‘had some effects on the decrease of the 24-hour urinary albumin excretion rate (UAER), 24-hour urinary protein, Scr, and BUN, and also on the improvement of Ccr’ (p. 727). They concluded that it ‘has some effect and is relatively safe in treating patients with DN’. Another study [17] evaluated the efficacy and safety of a TCM capsule, *Xuezhikang,* in the treatment of DN. The results of meta-analyses showed that it ‘was superior to routine treatment in decreasing 24-h urinary protein, microalbuminuria and UAER’. However, ‘due to a high risk of selection bias and detection bias in the included studies, the evidence is insufficient to determine the effect of *Xuezhikang*’.

Obviously, it is notable that most published clinical trials which assessed the efficacy of TCM on DN were of poor methodology, and, therefore, their results have been invalidated. It is necessary to carry out well-designed clinical trials to provide sound evidence. The present clinical study introduces those promising and classical herbs and formulae into the treatment of microalbuminuria. To make administration convenient, a TCM decoction will be processed in the form of granules. The present trial is a study of great value for it will provide important parameters for a future randomized, placebo-controlled, clinical trial with a large sample size.

### Trial status

Patient recruitment for the trial is on-going. Data collection will continue until the end of 2013.

## Abbreviations

ACE: Angiotensin converting enzyme; ACR: Albumin-to-creatinine ratio; ARB: Angiotensin II receptor blocker; BUN: Blood urea nitrogen; BP: Blood pressure; Ccr: Clearance of creatinine; DN: Diabetic nephropathy; FPG: Fasting plasma glucose; GFR: Glomerular filtration rate; HbA1c: Hemoglobin; Scr: Serum creatinine; TCM: Traditional chinese medicine; UAE: Urinary albumin excretion.

## Competing interests

Xiang Tu and Sen Zhong are currently applying for patents concerning *Flastem Milkvetch Seed* and *Huang Qi Elixir* formula for proteinuria/diabetic nephropathy. The other authors declare they have no competing interests.

## Authors’ contributions

XT and SZ conceived of the study. XT, FL, XFY, PF and FW designed the study. JBJ named the formula. XT and JBJ drafted the manuscript. All authors have read and approved the final manuscript.
